# Supplementary material for: Sub‐Picosecond Carrier Dynamics Explored using Automated High‐Throughput Studies of Doping Inhomogeneity within a Bayesian Framework
Source: Small. 2023 Apr 24;19(33):2300053. doi: 10.1002/smll.202300053 (PMC11475383; doi:10.1002/smll.202300053)
Supplement: Supplementary file 1 — Supporting Information [file SMLL-19-2300053-s001.pdf]

NANO small MICRO

Supporting Information

for *Small,* DOI: 10.1002/smll.202300053

Sub-Picosecond Carrier Dynamics Explored using Automated High-Throughput Studies of Doping Inhomogeneity within a Bayesian Framework

Ruqaiya Al-Abri, Nawal Al Amairi, Stephen Church, Conor Byrne, Sudhakar Sivakumar, Alex Walton, Martin H. Magnusson, and Patrick Parkinson**

Sub-Picosecond Carrier Dynamics Explored using Automated High-Throughput Studies of Doping Inhomogeneity within a Bayesian Framework

Ruqaiya Al-Abri,[*,†] Nawal Al Amairi,[†] Stephen Church,[†] Conor Byrne,[‡] Sudhakar Sivakumar,[¶] Alex Walton,[‡] Martin H. Magnusson,[¶] and Patrick Parkinson[†]

†*Department of Physics and Astronomy and the Photon Science Institute, University of Manchester, Oxford Road, Manchester, M13 9PL, United Kingdom*

‡*Department of Chemistry, University of Manchester, Oxford Road, Manchester, M13 9PL, United Kingdom*

¶*Department of Physics and NanoLund, Lund University, Box 118, SE-221 00, Sweden*

Supporting Information

1 X-Ray Photoelectron Spectroscopy (XPS) Measurements

XPS measurements were carried out on the Zn-doped GaAs NWs to determine the atomic concentration of the Zn dopant. The NWs were characterized as-deposited on silicon substrate with a native oxide. X-ray photoelectron spectroscopy (XPS) measurements were performed with SPECS XPS instrument, equipped with a SPECS Focus 500 monochromated Al Kα X-ray source with photon energy of 1486.6 eV and an argon-ion sputtering

Table S1: Summary of reference and Zn-doped GaAs NWs studied showing Zn flow, dopant level p from XPS and hole density from optical measurements from μ-PL spectroscopy.

Sample	Zn Flow	XPS (Ga:Zn)	XPS Zn density(cm^{-3})	Optical hole density (cm^{-3})
Undoped-GaAs	$\sim 0\,\%$	1:0.012	$0.26(11)\times10^{21}$	-
Zn-GaAs	$1.5\,\%$	1:0.060	$1.32(11)\times10^{21}$	$9.67^{17.76}_{5.08} \times 10^{19}\,cm^{-3}$

source. Emitted photoelectrons were collected using a 150 mm hemispherical energy analyzer (SPECS Phoibos 150). Detailed scans were recorded for Zn2p and Ga2p core levels at a pass energy of 30 eV. The areas of the peaks were corrected for the known relative sensitivity factors to calculate Zn:Ga concentration ratios.

The XPS spectrum is shown in Figure S1a. The sample shows photoemission peaks arising from Ga2p, As3s, and Zn2p which are used to determine the material stoichiometry by dividing the peak areas by their respective relative sensitivity factors for Al K alpha X-Rays. In addition, there are silicon and oxygen peaks associated with silicon oxide substrate and carbon due to the ambient exposure. Figure S1b and c show a magnified region at high energy attributed to Ga2P and Zn2p photoemission. Table S1 summarizes the main findings showing nominal Zn flow, the ensemble average of Zn level obtained from XPS, and the effective hole density derived from calculations based on the energy shift reported in the main text. The calculated dopant level using optical methods is noted to be around 20× lower than that determined from XPS. This may be due to an ensemble weighting effect, or more likely due to incomplete activation of Zn dopants at the high levels present as discussed in the manuscript.

2 Post-location Filtering of Nanowires

There is variation in photoluminescence emission due to inhomogeneity between individual wires, however, SEM imagery (main text) indicates that we also anticipate emission from clumps of multiple NWs and the presence of non-emissive dust or dirt. Following the initial NW location using dark-field optical microscopy, we use a filtering process to remove spectra

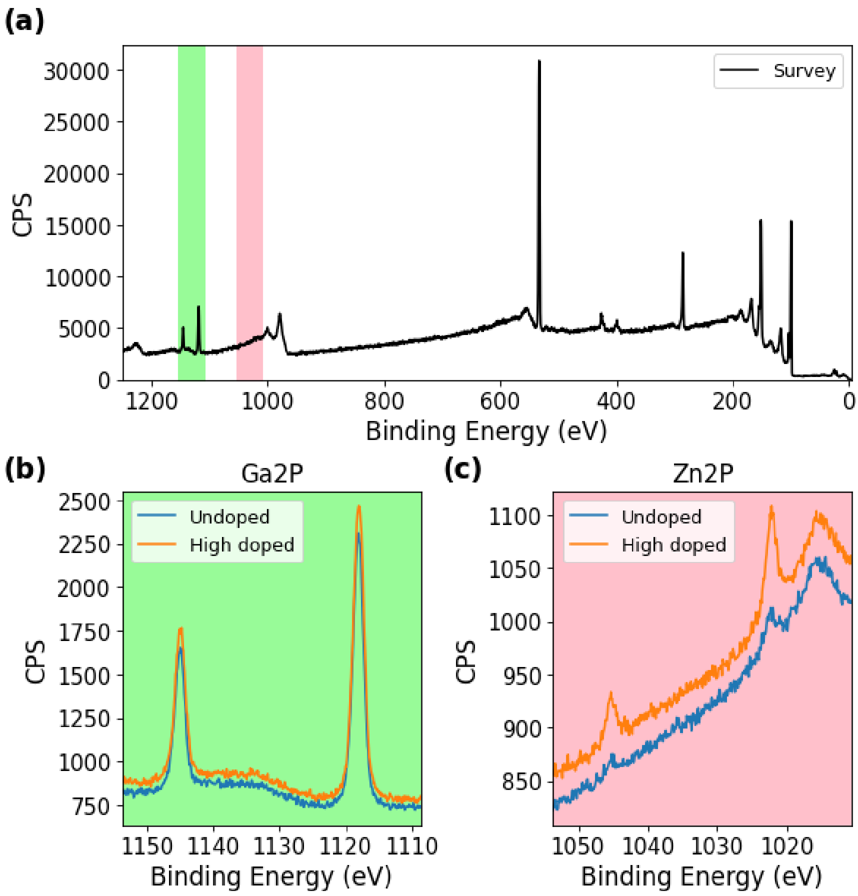

Figure S1: (Top) XPS survey spectra for Zn-doped GaAs on silicon oxide substrate. (Bottom) High-resolution XPS scans of the (left) gallium Ga2p and (right) zinc Zn2p regions for both nominally undoped and highly zinc-doped nanowires.

Table S2: Fitting parameters for the spectra shown in the main text showing peak energy, emission temperature, and emission amplitude for band-edge BE and split-off SO.

NW ID	BE energy (eV)	BE temperature (K)	BE amplitude	SO energy (eV)	SO temperature (K)	SO amplitude
17469	1.331	1093	1.10×10^{-17}	1.76	394	4.39×10^{-18}
17360	1.355	1051	2.15×10^{-17}	1.73	385	5.75×10^{-18}
13386	1.358	880	6.09×10^{-17}	1.73	321	1.05×10^{-17}
401	1.361	996	9.22×10^{-17}	1.72	305	1.88×10^{-17}

Table S3: Summary of filtering conditions of the output PL-model parameters with the number is wires removed. Some wires are removed by multiple filters - the numbers do not sum to the total removed.

Parameter	Condition	Justification	Wires removed
BE emission amplitude	$< 6 \times 10^{-16}$	Removing clumps	48
SO emission amplitude	$< 1.19 \times 10^{-16}$	Removing clumps	37
BE emission temperature	< 5450 K	Removing unphysical high temperatures	103
BE emission temperature	> 330 K	Removing unphysical low temperatures	962
SO emission temperature	< 700 K	Removing unphysical high temperatures	1928
SO emission temperature	> 305 K	Removing unphysical low temperatures	3216
BE peak energy	< 1.405 eV	Removing unphysical high energy	144
BE peak energy	> 1.301 eV	Removing unphysical low energy	9601
SO peak energy	< 1.779 eV	Range of SO emission is known	1037
SO peak energy	> 1.671 eV	Range of SO emission is known	2893

likely to be contaminants. The photoluminescence emission PL model output is used to create a threshold above which wires are defined as clumps, which are removed before further analyzing. In addition, the emission temperature and peak energy outside reliable range or representing physically unrealistic wires were removed. Examples of the fitting parameters are shown in Table S2 for the four spectra illustrated in the main text. Table S3 shows the benchmarks in which the parameters were filtered and the number of wires removed. This results in removing around 54% of the wires ending up with 11,487 wires.

3 Scaling of Internal Quantum Efficiency to Photoluminescence Intensity

The photoluminescence intensity PL is related to the internal quantum efficiency IQE by a scaling factor α and the diameter-dependent NW absorption ($A(d)$) as mentioned in the main text. Quantifying these factors is crucial in setting a prior for Bayesian analysis.

3.1 Experimental Factor α

The scaling factor α is related to experimental conditions during the acquisition of PL spectra namely laser power, laser spot size, objective lens collection efficiency, microscope throughput efficiency, and spectrometer quantum efficiency. All of these conditions were approximated experimentally by an end-to-end calibration of laser reflection from a mirror in the sample position.

3.2 NW Absorption Modelling

The NW absorption as a function of diameter was determined using COMSOL simulation. In the simulation, power loss density (PLD) was studied for a range of NW diameters from 10-200 nm under simulated excitation conditions to obtain the absorption percentage. The PLD was found for p- and s- polarized incident light, with light spot size 1 μm. The absorption was obtained by integrating the PLD across the NW length and normalising it to the incident power and spot diameter. Figure S2a depicts NW absorption of light with second-degree polynomial fit at different NW diameters, the shaded area indicates the range of diameter of interest. While a quadratic fit is naive, reflecting the increasing absorption with increasing geometric area presented to the beam and increasing thickness in the sub-absorption depth regime, the model does not pass through the data points. The NW model used in COMSOL reflects interference related to scattering and substrate interactions, which appear as oscillation. However, the error introduced is relatively small - on the order of $< 2\times$ - and we choose to neglect this in our data analysis. An example of NW absorption of light is shown in Figure S2b for s-polarized light at three different NW diameters.

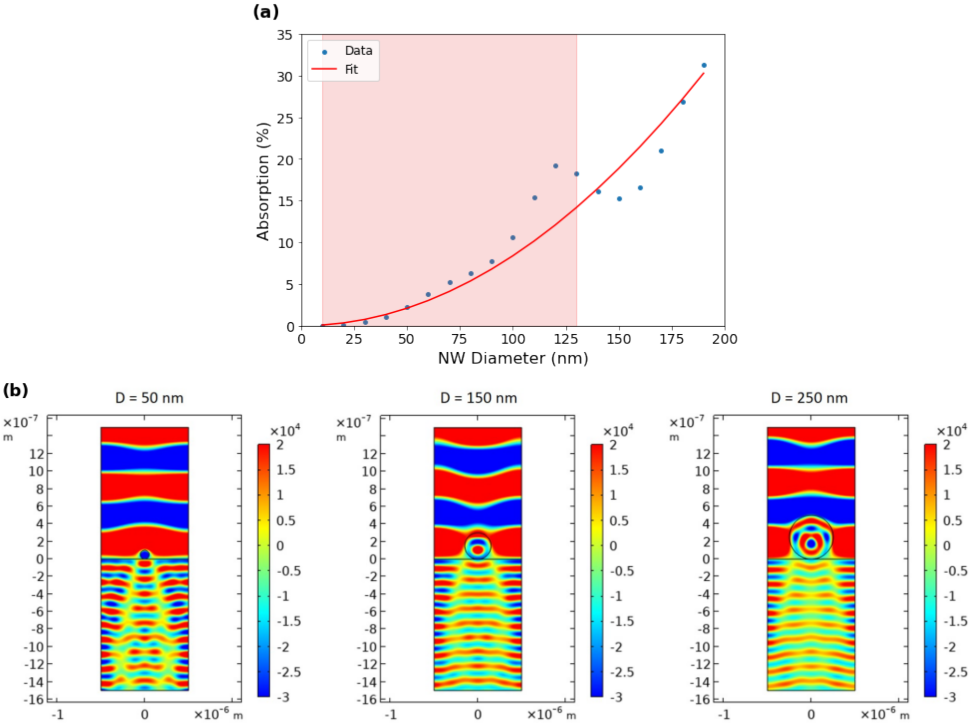

Figure S2: (a) Relation between NW absorption of light and diameter, the red line is a second-degree polynomial fit to the data. The shaded area illustrates the range of diameter of interest 10-130 nm. (b) Electric field distribution displaying light absorption of s-polarized light at three NW diameters 50, 150, and 250 nm.

4 Markov-Chain Monte-Carlo Model

4.1 Prior Probability Distribution Functions

Bayesian inference is a process of updating prior knowledge with new evidence or data. The prior vector Ψ is a list of probability distribution functions (PDFs) that represents our knowledge of each parameter before considering the data. In line with convention, we use analytical PDFs, with our choice of function depending on our confidence in each parameter; these are listed in the main text. The bandgap of GaAs E_0 is a well-known material parameter, hence the prior is set as a normal distribution $\mathcal{N}(\mu, \sigma)$ centred at 1.405 eV with a narrow full width at half maximum (FWHM) of 5 meV reflecting our small uncertainty. Equation 1 describes normal distribution where μ and σ are the mean and standard deviation of the distribution,

$$\mathcal{N}(\mu, \sigma) = e^{-0.5((x-\mu)/\sigma)^2}. \tag{1}$$

In some cases a range of values have been reported in the literature - for instance the radiative recombination coefficient B - or where we base our prior on physical upper and lower limits - for instance the doping level p. In this case, a generalized distribution $G(\mu, \sigma, \beta)$ is used, which is a normal distribution with higher uncertainty used

$$\mathcal{G}(\mu, \sigma, \beta) = e^{-0.5((x-\mu)/\sigma)^{\beta}}. \tag{2}$$

The parameter β represents the width of the prior distribution. Finally, in some cases hard upper and lower limits are known, such as for carrier initial lifetime. However, we have little further a-priori insight and the prior distribution is therefore uniform

$$\mathcal{U}(x_{min}, x_{max}) = (x >= x_{min}) \cap (x <= x_{max}). \tag{3}$$

In addition, these distributions may be combined or truncated to be positive to avoid physically unrealistic values. The distributions are schematically shown in Figure S3.

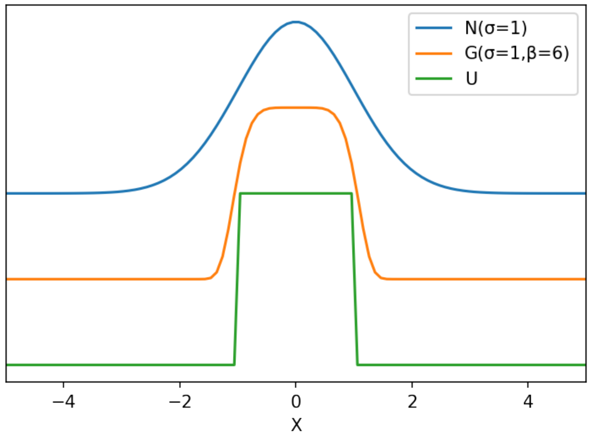

Figure S3: Schematic of three probability distribution functions centred at $x = 0$ with scale parameter $\sigma = 1$.

4.2 Posterior Probability Distributions

Figure S4 shows the distributions and correlation plots between all model posterior distributions as a function of doping $\log(p)$ and diameter d. As described in the main text, posterior samples are separated into regions with experimental support (evidenced regions) and the full range returned by the model. Additionally, samples with very high values of α are removed to improve the visualization of the correlations ($>2 \times 10^{-15}$) resulting in removing 7% of the sampling data-set. The figure illustrates no strong correlations between the parameters as a function of d and $\log(p)$ before and after masking; the correlation between $\log(p)$ and d is investigated in detail in the main text.

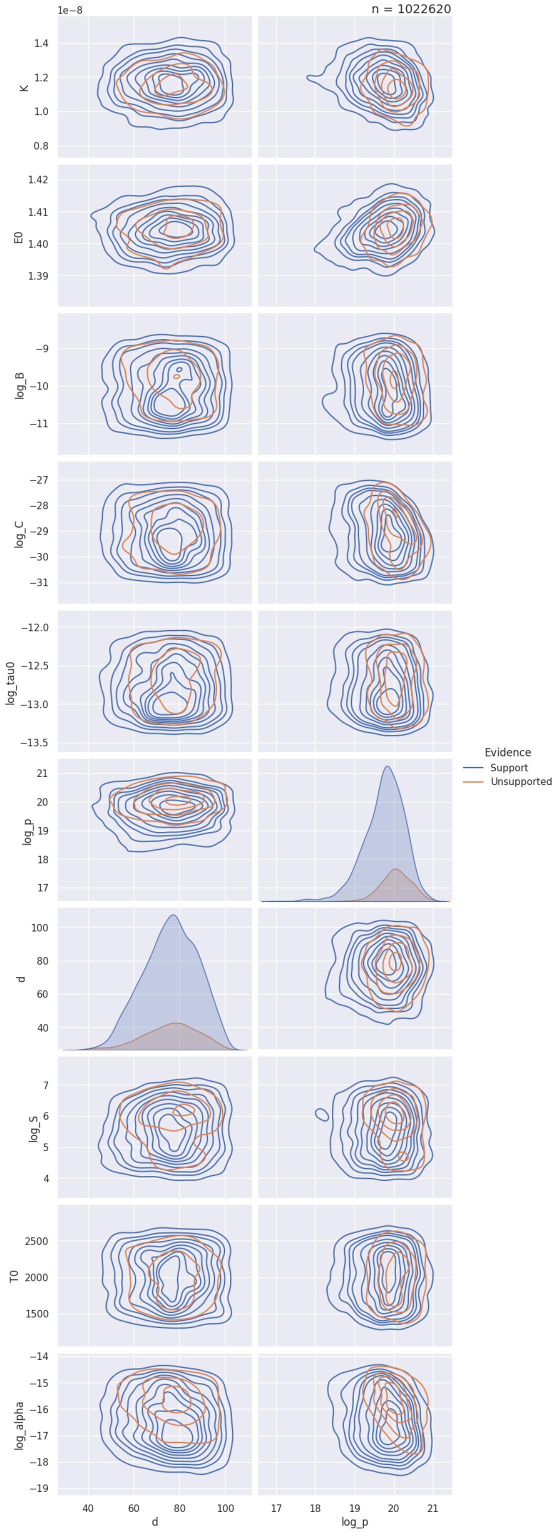

Figure S4: Pair posterior correlations for all the parameters determined using MCMC, including K, E_0, $\log(B)$, $\log(C)$, $\log(\tau_0)$, $\log(p)$, d, $\log(S)$, T_0, and $\log(\alpha)$ versus d and $\log(p)$. The number (n) at the top of the figure indicates the number of samples under investigation.

5 Fermi Energy Shift

The location of the Fermi level in a p-type doping semiconductor is proportional to the logarithmic hole density, as given in the following relation

$$\Delta E = K_B T \ln\left(\frac{N_V}{p}\right), \tag{4}$$

where ΔE is the location of the Fermi level with respect to the valence band-edge, $K_B T$ is thermal energy given as 25 meV, N_V is the density of state in the valence band given as $9 \times 10^{18}\,\mathrm{cm}^{-3}$, and p is the hole density. Given the spread in hole density, the shift of Fermi level is calculated for each doping measured as illustrated in Figure S5a. The negative values indicate that Fermi level is below the valence band. For a Fermi-level within the gap, no correction is needed. However, the doping regime above $1 \times 10^{19}\,\mathrm{cm}^{-3}$ leads to degenerate doping, and a correction to the effective emission temperature is required. The electron excess energy E^{e}_{exc} and hole excess energy E^{h}_{exc} due to the incident photo-excitation energy $E_{inc} = 1.96\,\mathrm{eV}$ are calculated using the hole-electron effective mass ratio $r_{m^*} = 0.118$ and the spread in the band-edge energy E_g, that is

$$E_{exc} = E_{inc} - E_g \tag{5}$$

$$E^{h}_{exc} = E_{exc} \times r_{m*} \tag{6}$$

$$E^{e}_{exc} = E_{exc} - E^{h}_{exc} \tag{7}$$

Figure S5b depicts the Fermi energy, electron, and hole excess energies with respect to doping. Fermi energy intercepts the hole excess energy at doping $1.5 \times 10^{20}\,\mathrm{cm}^{-3}$ revealing that above this doping level GaAs NWs absorption to the valance band will drop.

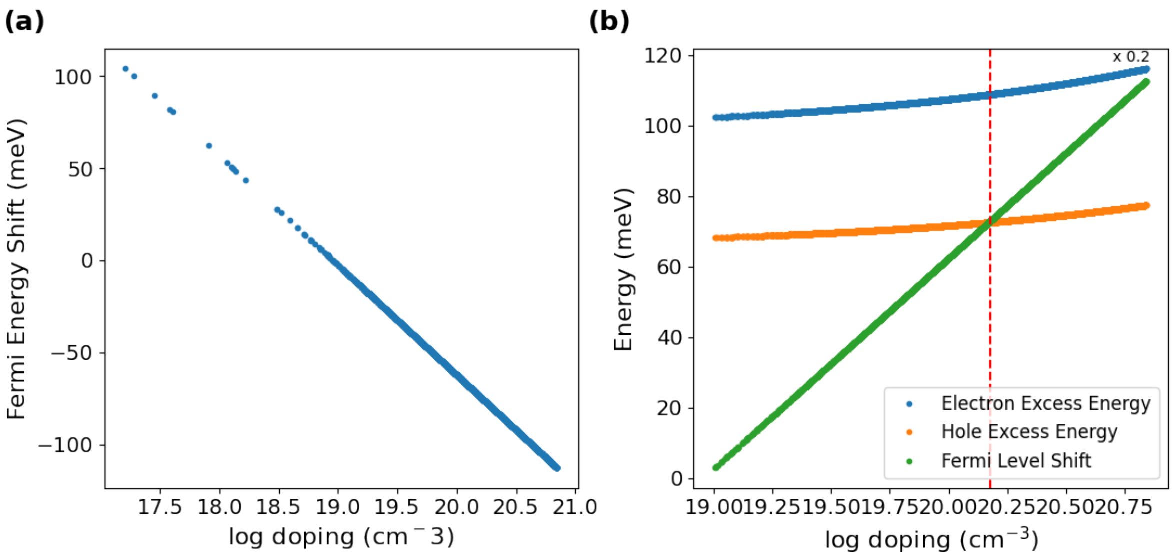

Figure S5: (a) Fermi energy shift as a function of logarithmic doping. (b) Hole excess energy and scaled electron excess energy versus doping compared to Fermi energy. The vertical line indicates the doping level beyond which GaAs NWs absorption into the valance band is affected.

5.1 Effective Carrier Temperature

Due to the spread in Fermi energy level shift, the photo-excited electrons can recombine with holes below the valence band edge, giving rise to hot emission. By eliminating the effect of Fermi temperature, the effective electron temperature from the band-edge and split-off are comparable as shown in Figure S6; with a gradient of $0.91_{0.90}^{0.92}$ K/K. This indicates that both band-edge and split-off recombination processes are likely to take place at the same timescale. Here we show only a high doping regime i.e., positive Fermi energy.

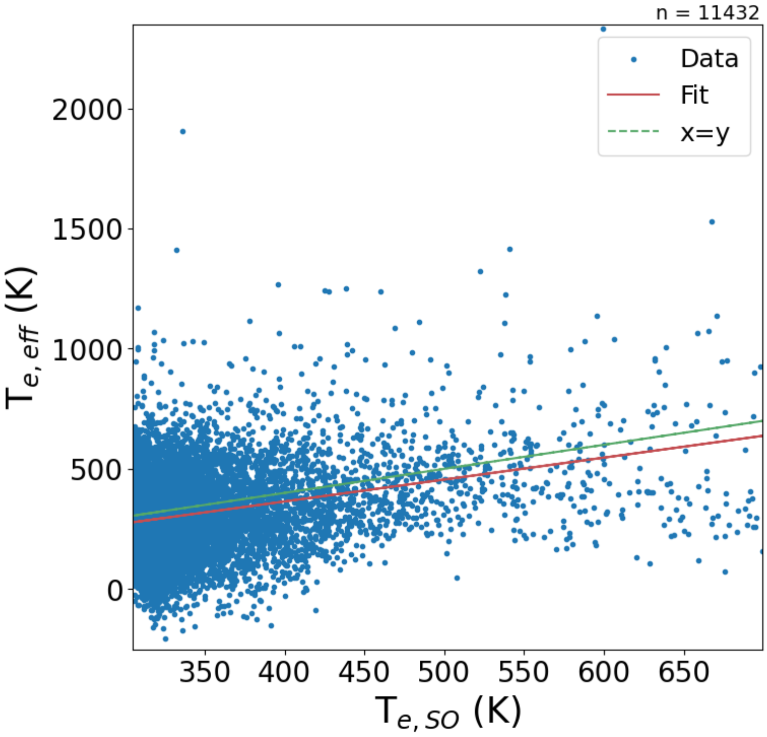

Figure S6: Comparison of effective electron temperature from the band-edge and the split-off temperature, at positive Fermi energies, showing a gradient of $0.91^{0.92}_{0.90}$K/K . The number (n) at the top of the figure indicates the number of samples under investigation. Using two-sided Pearson correlation between effective electron temperature from the band-edge and the split-off temperature with $p - value = 0$ and $\rho = 0.268$.
